# Urinary tract infections and reduced risk of bladder cancer in Los Angeles

**DOI:** 10.1038/sj.bjc.6604889

**Published:** 2009-01-27

**Authors:** X Jiang, J E Castelao, S Groshen, V K Cortessis, D Shibata, D V Conti, J-M Yuan, M C Pike, M Gago-Dominguez

**Affiliations:** 1 Department of Preventive Medicine, Keck School of Medicine, University of Southern California, Los Angeles, CA 90089, USA; 2 Department of Pathology, Keck School of Medicine, University of Southern California, Los Angeles, CA 90089, USA; 3 The Masonic Cancer Center, University of Minnesota, Minneapolis, MN 55454, USA

**Keywords:** bladder cancer, urinary tract infection, bladder infection, Los Angeles, antibiotics

## Abstract

We investigated the association between urinary tract infections (UTIs) and transitional cell carcinoma of the bladder in a population-based case–control study in Los Angeles covering 1586 cases and age-, gender-, and race-matched neighbourhood controls. A history of bladder infection was associated with a reduced risk of bladder cancer among women (odds ratio (OR), 0.66; 95% confidence interval (CI), 0.46–0.96). No effect was found in men, perhaps due to power limitations. A greater reduction in bladder cancer risk was observed among women with multiple infections (OR, 0.37; 95% CI, 0.18–0.78). Exclusion of subjects with a history of diabetes, kidney or bladder stones did not change the inverse association. A history of kidney infections was not associated with bladder cancer risk, but there was a weak association between a history of other UTIs and slightly increased risk among men. Our results suggest that a history of bladder infection is associated with a reduced risk of bladder cancer among women. Cytotoxicity from antibiotics commonly used to treat bladder infections is proposed as one possible explanation.

Urinary tract infections (UTIs) are the most common kidney and urologic diseases in the US, especially among women. About 50% of women have at least one symptomatic infection during their lifetime, and many have recurrent episodes (Stamm, 2002). However, UTIs in men are uncommon until after age 50, and are usually indicative of an underlying urologic abnormality (Stamm and Hooton, 1993). In the US, the majority of uncomplicated UTIs are caused by *Escherichia coli* (80%) or *Staphylococcus saprophyticus* (10–15%) (Stamm, 2002). An experimental study in rats has shown associations between chronic infections with *Escherichia coli* and early bladder neoplasia (Davis *et al*, 1991), and it has been suggested that the high rates of bladder cancer in some parts of Africa and the Middle East may be due to the high prevalence of chronic infection with *Schistosoma haematobium* (IARC, 1994). A review of published epidemiological studies regarding the association between bladder infections and transitional cell carcinoma (TCC), the predominant type in developed countries, shows inconsistent results (Howe *et al*, 1980; Kantor *et al*, 1984; Claude *et al*, 1986; Piper *et al*, 1986; Kjaer *et al*, 1989; Gonzalez *et al*, 1991; La Vecchia *et al*, 1991; Sturgeon *et al*, 1994; Jhamb *et al*, 2007) and reveals a need for future studies to determine whether UTIs increase the risk of bladder cancer.

We collected detailed information on UTIs in a large population-based case–control study of bladder cancer in Los Angeles County, where we have identified risk factors including cigarette smoking (Castelao *et al*, 2001) and use of permanent hair dyes among women (Gago-Dominguez *et al*, 2001, 2003), and protective factors including use of non-steroidal anti-inflammatory drugs (NSAIDs) (Castelao *et al*, 2000) and consumption of carotenoids and vitamin C (Castelao *et al*, 2004).

## Materials and methods

Cases were identified through the Los Angeles County Cancer Surveillance Program – the largest of the National Cancer Institute's Surveillance, Epidemiology and End Results (SEER) cancer registries (Bernstein and Ross, 1991). Eligibility criteria included histologically confirmed bladder cancers diagnosed between January 1, 1987 and April 30, 1996 among non-Asians between the ages of 25 and 64 years. In total, 2384 cases were identified. Of these, 210 (9%) died before we could contact them or were too ill to be interviewed. Permission to contact 99 (4%) patients was denied by their physicians, and 404 (17%) patients declined to participate in the study. We interviewed 1671 (70%) of the cases.

For each enrolled case, a standard procedure was followed to recruit a control subject from the neighbourhood of the residence of the case at the time of cancer diagnosis, with the control matched to the case by age (within 5 years), sex, and race (non-Hispanic white, Hispanic white, or African American/others). We attempted to identify the age, sex, and race of all inhabitants of each housing unit; ‘not at home’ units were systematically revisited to complete the census. The first resident along this defined route who satisfied all eligibility criteria for controls was asked to participate in this study (i.e. first eligible control). If that individual refused, the next eligible control (i.e. second eligible control) in the sequence was asked and so on until we located an eligible control who agreed to be interviewed. When we failed to find any resident who met our matching criteria after canvassing 150 housing units, we excluded race from the matching criteria. If a matched control subject based on this relaxed criterion could not be found within a maximum of 300 housing units, the case was dropped from the study. For the 1671 interviewed patients, a matched control could not be found for 5% (85) of the interviewed cases. Among the 1,586 eligible control subjects, 1090 (69%) control subjects were first eligible controls, 325 (20%) were second eligible controls and 111 (7%) were third eligible controls, and the remaining 60 (4%) control subjects were fourth or higher eligible controls. Twenty-one control subjects were not matched to the index case by race. Therefore, a total of 1586 pairs of cases and controls were included in the analyses. All study subjects signed informed consent forms. The study was approved by the Institutional Review Board at the University of Southern California, Keck School of Medicine.

All study cases and controls were interviewed at home by trained interviewers using a structured questionnaire. The structured questionnaire requested information up to 2 years before diagnosis of cancer for the cases, and up to 2 years before diagnosis of cancer of the index case for the matched controls (i.e. reference year). Each subject was asked to report information on demographic characteristics, lifetime use of tobacco products and alcohol, usual adult dietary habits, lifetime occupational history, prior medical conditions, and prior use of medications. The questionnaire included questions on history of physician-diagnosed hypertension, diabetes, angina, heart attack, stroke, analgesic nephropathy, renal papillary necrosis, kidney/renal stones, bladder stones, polycystic kidney disease, hereditary kidney disease, and injury to kidney. After January 1992, a history of tuberculosis, thyroid disease, polycythemia vera, and gout was also asked. For each medical condition, we asked the year of first diagnosis by a doctor. In addition, we asked the participants if they were told by a doctor that they had kidney infections, bladder infections, or other type of UTIs. If so, the year of first and last diagnosis and total number of diagnoses were also requested.

### Statistical analysis

The associations of bladder cancer with medical histories were measured by odds ratios (ORs) and their corresponding 95% confidence intervals (CIs) and *P*-values. Conditional logistic regression models were used to examine the relationship between any medical history and bladder cancer risk with adjustment for the following risk factors for bladder cancer: number of cigarettes smoked per day, number of years of smoking, smoking status in the reference year (smoker or nonsmoker) (Castelao *et al*, 2001), level of education (high school or less, some college, college or above), total number of NSAID pills used over the lifetime (non/irregular user, <1441 pills, ⩾1441 pills used over lifetime) (Castelao *et al*, 2000), intake of carotenoids (quintiles) (Castelao *et al*, 2004), and duration of employment as a hairdresser/barber (years) (Castelao *et al*, 2001). There were no material changes in the results with further adjustment for hair dye use, and thus this variable was not retained. Unconditional logistic regression was used in the analyses stratified by smoking status (lifetime nonsmokers *vs* ever smokers). In addition to all the covariates mentioned above, strata defined by age and sex (age groups of <46, 46–50, 51–55, 56–60, and >60 years for each sex), and racial/ethnic groups (non-Hispanic white, Hispanic white, or African American/others) were included in the regression model to account for the matched sampling. All results were similar when we limited our analyses to non-Hispanic whites only, or when we excluded from our analyses the case–control pairs that were not matched on race. Owing to this, and in order to maximize our study power, all study subjects were included in our analyses.

Statistical analyses were performed using the SAS version 9.1 (SAS Institute Inc., NC, USA). ORs with two-sided *P*-values <0.05 were considered statistically significant. All *P*-values, estimated from likelihood ratio test, were two-sided.

## Results

A history of one or more UTIs was reported by 26% of cases and 28% of controls (Table 1), occurring more frequently in women (47% of cases and 57% of controls) than in men (20% of cases and 20% of controls). In women, the most frequent UTIs were bladder infections; in men, however, UTIs occurred more frequently in urinary tract organs other than the kidney and bladder, presumably in the urethra and ureter. Thus, among the 200 control women reporting a history of UTI, 169 (85%) reported a history of bladder infections, 43 (22%) reported kidney infections, and only 18 reported (9%) other UTIs. The corresponding figures among the 242 control men reporting a history of UTI were 100 (41%), 52 (21%), and 115 (48%), respectively.

A history of any UTI was not associated with bladder cancer among all subjects, men and women combined (OR, 1.00; 95% CI, 0.83–1.20; Table 1). Women reporting a history of UTI had a 26% reduction in bladder cancer risk (OR, 0.74; 95% CI, 0.53–1.03) than women reporting no history of any UTI; however, this reduction did not reach statistical significance. A history of kidney infection or other UTIs was not significantly associated with bladder cancer; however, the risk was slightly elevated at borderline significance level among men reporting a history of other UTIs (OR, 1.35; 95% CI, 0.99–1.83), especially among those reporting infection occurring within 5 years of cancer diagnosis (within 5 years of cancer diagnosis: OR, 3.01; 95% CI, 1.26–7.23; ⩾5 years before cancer diagnosis: OR, 1.17; 95% CI, 0.83–1.64).

Bladder infections were much more frequent among women than among men in our study (48 *vs* 8%, respectively). Overall, a history of bladder infection was associated with a slightly reduced risk of bladder cancer (OR, 0.80; 95% CI, 0.63–1.02; Table 1), although the association was not statistically significant. This inverse association was, however, seen only in women (OR, 0.66; 95% CI, 0.46–0.96). Exclusion of women with a bladder infection within 5 years of cancer diagnosis strengthened the inverse association (OR, 0.52; 95% CI, 0.34–0.81; Table 2), suggesting that the observed inverse bladder infection–bladder cancer association was unlikely to be caused by bladder infections resulting from the bladder cancer. Thus, all subsequent analyses were restricted to women who did not have a history of recent bladder infections. We also assessed the association between number of episodes of bladder infections and bladder cancer risk. The largest reduction in risk was observed in women with more than three episodes of infections (OR, 0.37; 95% CI, 0.18–0.78; Table 2). We also explored the potential modifying effect of age at first and last infection in our observed inverse association, although the data were too sparse to make any strong conclusions. Among women who did not report a history of recent bladder infections, the reported median ages at first and last infection were 29 and 37 years, respectively. The infection-associated reduction in risk seemed to be similar between women who had their first episode of bladder infection at a younger age (<29) and those who had their first episode of bladder infection at an older age (⩾29) (*P*_heterogeneity_=0.76). Similarly, no significant differences in risk were observed by age at last infection (*P*_heterogeneity_=0.81).

The inverse association between bladder infections and bladder cancer was observed in all categories of smoking (Table 3), although the association was particularly strong among former smokers (*P*_⩾1 *vs* 0_=0.002). The OR associated with a history of multiple bladder infections (>3) was 0.43 (95% CI, 0.11–1.68) among nonsmokers, 0.21 (0.07–0.68) among former smokers, and 0.69 (0.20–2.39) among current smokers.

In our study population, control subjects with a history of bladder infection were more likely to use NSAIDs (42.7%) than those without such a history (33.3%, *P*=0.004). Therefore we further analysed data stratified by the use of NSAIDs. The inverse association between bladder infection and bladder cancer among women was present in both users (OR, 0.54; 95% CI, 0.29–1.03) and non-users of NSAIDs (OR, 0.52; 95% CI, 0.31–0.87), with a *P*-value of 0.87 for the interaction between use of NSAIDs and bladder infection on cancer risk (data not shown).

To investigate whether the bladder infection-bladder cancer association varied across strata defined by other known risk/protective factors in this study, including age, level of education (Jiang *et al*, 2007), alcohol consumption (Jiang *et al*, 2007), water intake (Jiang *et al*, 2008), and intake of carotenoids (Castelao *et al*, 2004), stratified models were fit according to the levels of those variables. An inverse association between bladder infection and bladder cancer was observed in virtually all subgroups (data not shown).

Regarding grade and stage of disease, a history of bladder infection was significantly associated with a reduced risk of only invasive (T1–T4) bladder cancers (*P*_⩾1 *vs* 0_=0.005; Table 4), but not non-invasive (Ta) bladder cancers (*P*_⩾1 *vs* 0_=0.11). In addition, the protective effect differed by grade, with the effect being more pronounced for high-grade (grade 3–4) cancers (*P*_⩾1 *vs* 0_=0.0004).

A history of diabetes was associated with an increased risk of bladder cancer (OR, 1.70; 95% CI, 1.18–2.45), whereas a history of kidney stones was associated with a slight increase in bladder cancer risk (OR, 1.24; 95% CI, 0.94–1.64). A history of bladder stones, which was reported only by very few subjects (10 cases and 11 controls), was not associated with bladder cancer risk (OR, 0.79; 95% CI, 0.30–2.10). The inverse association with bladder infections was materially unchanged after excluding subjects with a history of diabetes, kidney, and/or bladder stones.

## Discussion

UTIs are very common among women (Stamm, 2002), but uncommon among men until after the age of 50 years (Stamm and Hooton, 1993). Bladder infection is the most common UTI among women in the US (Sheffield and Cunningham, 2005). Our study found a significant association between bladder infections and reduced risk of bladder cancer among women, with the highest protection found among women who experienced multiple infections. This inverse association was confined to invasive high-grade bladder cancers.

A number of case–control studies have examined the possible association of UTIs with bladder cancer in developed countries (Howe *et al*, 1980; Kantor *et al*, 1984; Claude *et al*, 1986; Piper *et al*, 1986; Kjaer *et al*, 1989; Gonzalez *et al*, 1991; La Vecchia *et al*, 1991; Sturgeon *et al*, 1994; Jhamb *et al*, 2007). The overall evidence supports an increased risk from UTIs. However, in general, a much higher risk was observed for infections occurring approximately within 5 years of cancer diagnosis, and the risk was substantially reduced or disappeared when infections occurred many years before cancer diagnosis (Howe *et al*, 1980; Gonzalez *et al*, 1991; La Vecchia *et al*, 1991; Jhamb *et al*, 2007). As the dates of UTIs were not obtained in all these studies, it is also possible that the occurrence of UTIs may be a consequence of early bladder cancer before its diagnosis, rather than a cause of the disease. Alternatively, UTIs may have a later-stage effect in bladder carcinogenesis by promoting initiated tumors (La Vecchia *et al*, 1991). In addition, studies that found increased risks from UTIs (Claude *et al*, 1986; Kantor *et al*, 1988; Gonzalez *et al*, 1991) did not discriminate between the different types of UTI. This is an important consideration, as different types of UTI may have different impacts on bladder cancer risk. A history of cystitis has been reported to be associated with an increased bladder cancer risk, but a history of kidney infection was associated with a significantly decreased risk (Jhamb *et al*, 2007). In our study, a decreased risk of bladder cancer for bladder infections was found among women, but an increased risk for infections occurring in urinary tract organs other than kidney and bladder, presumably urethra, was found in men. The majority of urethritis in men are secondary to sexually transmitted diseases such as chlamydia and gonorrhoea (Wong and Stamm, 1983), which has been found to be associated with a significantly increased risk in three studies (Mommsen and Sell, 1983; La Vecchia *et al*, 1991; Michaud *et al*, 2007). If a history of UTI indeed increased the risk, one would expect a higher incidence of bladder cancer among women than among men, as well as a higher incidence of squamous cell carcinoma of the bladder *vs* TCC (Burin *et al*, 1995). However, bladder cancer is 3–4 times more common in men than in women, and approximately 90% of bladder cancer in the US are TCCs (with only 7% being of squamous cell carcinoma type) (Yu and Ross, 2002). Human and experimental studies have shown that bladder infections induce squamous metaplasia of the bladder, a process that may subsequently lead to squamous cell carcinoma of the bladder, but not to TCC (Burin *et al*, 1995), which is also supported by the strong associations between bladder infections and squamous cell carcinomas of the bladder, and not with TCC (IARC, 1994; Burin *et al*, 1995; Michaud, 2007).

Our study found a significant association between bladder infections, especially multiple infections, and reduced risk of bladder cancer. There are several possible reasons to explain these findings: (1) the anti-cancer effect of antibiotics used to treat bladder infections; (2) the higher intake of NSAIDs among subjects with a history of bladder infection; and (3) the immune response triggered by bladder infection.

In experimental studies, antibiotics used to treat bladder infections have been found to inhibit bladder cancer growth (Seay *et al*, 1996; Ebisuno *et al*, 1997; Aranha *et al*, 2000, 2002; Kamat and Lamm, 2004). The traditional treatment of uncomplicated UTIs has been a short course of antibiotics such as amoxicillin, trimethoprim (TMP) alone or with sulfamethoxazole (TMP-SMX), nitrofurantoin, cephalosporins, and fosfomycin (Stamm, 2002). Antibiotic agents preferred for UTIs are agents that are primarily excreted through the urinary tract and achieve prolonged and high concentrations in the urine (Stamm, 2002). About 70–90% of TMP and 30% of SMX are excreted unchanged in the urine (Masters *et al*, 2003). Besides their bactericidal effect, the commonly used antibiotics TMP-SMX, cefazolin (cephalosporin), and nitrofurantoin significantly inhibited cell proliferation in human TCC cell lines (Seay *et al*, 1996), producing a proliferation inhibition rate as high as 95%. Inhibition occurred in a dose-dependent manner at concentrations attainable in the urine after oral administration (Kamat and Lamm, 2004). Similar dose–response anti-cancer effects were shown for ciprofloxacin or other fluoroquinolones (Seay *et al*, 1996; Ebisuno *et al*, 1997; Kamat and Lamm, 2004). The mechanism for the antibiotics’ cytotoxicity against bladder cancer cells remains unclear. Ciprofloxacin has been shown to cause cell-cycle arrest, disruption of calcium homeostasis, mitochondrial swelling, and redistribution of Bax (a pro-apoptotic protein) to the mitochondrial membrane, eventually leading to apoptosis in human TCC lines (Aranha *et al*, 2000, 2002).

As subjects with a history of bladder infection were more likely to use NSAIDs regularly than subjects without such a history, and intake of NSAIDs was earlier found to be negatively associated with bladder cancer in this population (Castelao *et al*, 2000), the inverse association risk was plausibly the result of excessive use of NSAIDs among subjects with a history of bladder infection. However, the protective effect of bladder infection was observed among both regular users and non- or irregular users of NSAIDs; therefore, the use of NSAIDs was unlikely to explain our observed protective effect.

Another mechanism that may explain our finding is the anti-cancer effect of the immune response triggered by bladder infections. BCG immunotherapy for bladder cancer has been widely accepted for its effect in decreasing recurrence rates and in increasing median time to recurrence (Alexandroff *et al*, 1999). BCG infection induces release of chemokines/cytokines, attracts and activates neutrophils, macrophages and T-cells, resulting in improved recognition and killing of tumor cells (Alexandroff *et al*, 1999). Bladder infection, which is mostly caused by *E. coli* in humans, may trigger an immune response similar to BCG treatment, killing hyperplastic cells before they transform into cancerous cells (Zasloff, 2007).

The infection–cancer association was modified by frequency of bladder infections, with the highest reduction in risk confined to women with ⩾2 bladder infections. An estimated 20–30% of women with a first UTI will have recurrent episodes, and 5% will have chronic recurrent infections (Foxman *et al*, 2000). For women who have recurrent infections, a low-dose antimicrobial prophylaxis taken daily or thrice weekly for 3–6 months is recommended to prevent recurrent infections (Stamm and Hooton, 1993; Fihn, 2003). Dosage seems to play a role in the anticancer effect of antibiotics, as the cancer-killing effect has been shown to be irreversible only at high doses (i.e. 250 *μ*g ml^–1^ ciprofloxacin) (Zehavi-Willner and Shalit, 1992).

Compared with early studies, we were able to obtain the age at first and last infection and were therefore able to identify that the infection was not likely a result of bladder cancer and, that detection bias could not explain our results. We were also able to exclude subjects reporting a history of diabetes, kidney stones, and bladder stones and therefore eliminate the possibility that the effect of bladder infections could be so explained. Some of our subgroup analyses, especially those conducted among women with specific smoking status or histology, were based on very small numbers; so interpretation must be cautious. We also had power limitations in such groups, as infection before age 20 or after age 49, which would have been potentially interesting with greater numbers. Thus, in order to ensure proper study power, median ages were used as cut-off points. Also, information on antibiotic therapy associated with UTIs and other diseases was not requested. Recall bias (i.e. the possibility that cancer patients were more likely to recall infections than healthy controls) has probably not played an important role, as the associations we observed are inverse. However, confounding may still have resulted from unmeasured socioeconomic, genetic, and other lifestyle factors that differentiate women who had infection from those who did not. Bladder infection may be a marker of a greater susceptibility to all kinds of infections and subsequently a greater likelihood of being prescribed antibiotics. Lastly, bladder cancer patients with a history of infection may be more likely to die, be too ill to be interviewed, or decline participation in our study, thus leading to potential selection bias, which could have explained some of our observed associations.

Our results suggest that a history of bladder infection is associated with a reduced risk of bladder cancer. Cytotoxicity against bladder cancer cells from antibiotics commonly used to treat bladder infections is proposed as a possible mechanism to explain this association.

## Figures and Tables

**Table 1:** Urinary tract infections and risk of bladder cancer by sex

	**All**		**Men**		**Women**	
**History of**	**Cases/controls**	**OR^a^ (95% CI)**	**Cases/controls**	**OR^a^ (95% CI)**	**Cases/Controls**	**OR^a^ (95% CI)**
*Any urinary tract infection*						
No	1172/1144	1.00	988/995	1.00	184/149	1.00
Yes	414/442	1.00 (0.83–1.20)	249/242	1.15 (0.92–1.44)	165/200	0.74 (0.53–1.03)
						
*Bladder infection* b						
No	1365/1317	1.00	1148/1137	1.00	217/180	1.00
Yes	221/269	0.80 (0.63–1.02)	89/100	0.96 (0.69–1.35)	132/169	0.66 (0.46–0.96)
						
*Kidney infection* b						
No	1480/1491	1.00	1179/1185	1.00	301/306	1.00
Yes	106/95	1.10 (0.80–1.52)	58/52	1.01 (0.65–1.55)	48/43	1.26 (0.76–2. 07)
						
*Other urinary infection* b						
No	1443/1453	1.00	1108/1122	1.00	335/331	1.00
Yes	143/133	1.28 (0.96–1.70)	129/115	1.35 (0.99–1.83)	14/18	0.76 (0.33–1.75)

a Conditional logistic regression, adjusted for level of education, use of NSAIDs, intake of carotenoids, number of years as a hairdresser/barber, cigarette-smoking status, duration of smoking, and intensity of smoking.

b Additional adjustment for infections at the other two sites did not materially change the associations.

**Table 2:** Bladder infections and risk of bladder cancer in women

	**Cases/controls**	**OR^a^ (95% CI)**
*History of bladder infection*		
No	217/180	1.00 (reference)
Yes	132/169	0.66 (0.46–0.96)
*P*_yes *vs* no_ b		0.027
		
*Duration between last infection and cancer diagnosis (years)*		
Unknown	23/20	0.82 (0.38–1.76)
<5	39/39	1.03 (0.55–1.93)
⩾5	70/110	0.52 (0.34–0.81)
*P*_yes (⩾5) *vs* no_ b		0.003
		
*Number of episodes of infection* c		
1	30/40	0.65 (0.34–1.24)
2–3	23/36	0.54 (0.28–1.07)
>3	17/34	0.37 (0.18–0.78)
*P*_trend_ d		0.29
		
*Age at first infection (years)* c ^,^ e		
<29 (median)	35/51	0.52 (0.30–0.92)
⩾29	34/58	0.51 (0.29–0.90)
*P*_heterogeneity_ d		0.76
		
*Age at last infection (years)* c		
<37 (median)	36/54	0.52 (0.29–0.92)
⩾37	34/56	0.53 (0.30–0.93)
*P*_heterogeneity_ d		0.81

a Conditional logistic regression, adjusted for level of education, use of NSAIDs, intake of carotenoids, number of years as a hairdresser/barber, cigarette-smoking status, duration of smoking, and intensity of smoking.

b Likelihood ratio test for history of bladder infection (yes *vs* no).

c Women with a bladder infection occurring within 5 years of cancer diagnosis were excluded.

d Subjects without a history of bladder infection were removed for purposes of calculating *P*-values based on the likelihood ratio tests for trend or heterogeneity.

e Data on age at first infection were missing for one case and one control.

**Table 3:** Bladder infections and risk of bladder cancer in women by cigarette smoking

**No. of episodes of bladder infection^a^**	**Cases/controls**	**OR (95% CI)**
*Non-smokers* b		
0	43/88	1.00
⩾1	14/44	0.63 (0.30–1.33)
*P*_⩾1 *vs* 0_ c		0.20
1	7/17	0.79 (0.29–2.16)
2–3	4/14	0.62 (0.18–2.13)
>3	3/13	0.43 (0.11–1.68)
*P*_trend_ d		0.42
		
*Smokers* e		
0	174/92	1.00
⩾1	56/66	0.52 (0.33–0.83)
*P*_⩾1 *vs* 0_ c		0.005
1	23/23	0.61 (0.31–1.20)
2–3	19/22	0.50 (0.25–1.01)
>3	14/21	0.45 (0.21–0.97)
*P*_trend_ d		0.57
		
*Former smokers* f		
0	54/47	1.00
⩾1	21/44	0.35 (0.17–0.73)
*P*_⩾1 *vs* 0_ c		0.002
1	10/14	0.58 (0.21–1.60)
2–3	5/14	0.31 (0.09–1.06)
>3	6/16	0.21 (0.07–0.68)
*P*_trend_ d		0.19
		
*Current smokers* f		
0	119/45	1.00
⩾1	35/22	0.61 (0.31–1.21)
*P*_⩾1 *vs* 0_ c		0.18
1	13/9	0.46 (0.17–1.29)
2–3	14/8	0.73 (0.27–1.96)
>3	8/5	0.69 (0.20–2.39)
*P*_trend_ d		0.64

a Women with a bladder infection occurring within 5 years of cancer diagnosis were excluded from the analyses.

b Unconditional logistic regression, adjusted for age, race, level of education, use of NSAIDs, and intake of carotenoids.

c Likelihood ratio test for history of bladder infection (⩾1 *vs* 0).

d Subjects without a history of bladder infection were removed for purposes of calculating P-values based on the likelihood ratio tests for trend.

e Unconditional logistic regression, adjusted for age, race, level of education, use of NSAIDs, intake of carotenoids, cigarette-smoking status, duration of smoking, and intensity of smoking. Due to sample size limitation, number of years as a hairdresser/barber was excluded from regression model.

f One case with unknown smoking status at reference year was excluded from the analyses.

**Table 4:** Bladder infections and risk of bladder cancer in women by histology

**No. of episodes of bladder infection^a^**	**Cases/controls**	**OR^b^ (95% CI)**
*Stage* c		
Ta		
0	117/102	1.00
⩾1	43/60	0.70 (0.39–1.25)
*P*_⩾1 *vs* 0_ d		0.11
1	20/26	0.81 (0.37–1.78)
2–3	13/15	0.74 (0.29–1.92)
>3	10/19	0.53 (0.21–1.31)
*P*_trend_ e		0.71
*T1–T4*		
0	93/70	1.00
⩾1	22/42	0.24 (0.09–0.62)
*P*_⩾1 *vs* 0_ d		0.005
1	9/11	0.41 (0.10–1.62)
2–3	8/18	0.26 (0.06–1.04)
>3	5/13	0.05 (0.004–0.52)
*P*_trend_ e		0.22
		
*Grade* c		
Grade 1–2		
0	148/129	1.00
⩾1	55/76	0.72 (0.44–1.20)
*P*_⩾1 *vs* 0_^d^		0.11
1	25/30	0.93 (0.46–1.89)
2–3	17/23	0.64 (0.29–1.44)
>3	13/23	0.56 (0.24–1.32)
*P*_trend_ e		0.46
Grade *3–4*		
0	67/48	1.00
⩾1	12/31	0.07 (0.02–0.34)
*P*_⩾1 *vs* 0_ d		0.0004
1	4/8	0.06 (0.004–0.98)
2–3	5/12	0.13 (0.02–1.05)
>3	3/11	0.03 (0.002–0.49)
*P*_trend_ e		0.47

a Women with a bladder infection occurring within 5 years of cancer diagnosis were excluded from the analyses.

b Conditional logistic regression, adjusted for level of education, use of NSAIDs, intake of carotenoids, number of years as a hairdresser/barber, cigarette-smoking status, duration of smoking, and intensity of smoking.

c Six cases with unknown stage, eleven Tis cases, seven cases with unknown grade, and their matched controls were excluded from the relevant analyses.

d Likelihood ratio test for history of bladder infection (⩾1 *vs* 0).

e Subjects without a history of bladder infection were removed for purposes of calculating P-values based on the likelihood ratio tests for trend.
